# IP_3_ 3-kinase B prevents bone marrow failure

**DOI:** 10.18632/oncotarget.4480

**Published:** 2015-06-15

**Authors:** Stephanie Rigaud, Karsten Sauer

**Affiliations:** Department of Immunology and Microbial Science, The Scripps Research Institute, La Jolla, CA, USA

The Greek philosopher Plato emphasized the importance of moderating personal desires for the functioning of society. The virtue of moderation is also critical for the live-long function of hematopoietic stem cells (HSC), the origin of all blood cells. To prevent undue activation, which can deplete HSC and increase the risk of blood cancer, HSC need to restrain signaling by phosphoinositide 3-kinases (PI3K) - a pivotal pathway whose hyperactivation contributes to many diseases [1]. Yet, the precise mechanisms limiting PI3K-signaling within HSC remain ill understood. We recently presented evidence that they include non-canonical PI3K-regulation by the little-studied enzyme inositoltrisphosphate 3-kinase B (IP_3_ 3-kinase B/Itpkb) in HSC [2]. *Itpkb^−/−^* mice died with HSC/hematopoietic-progenitor-cell (HPC) depletion and anemia. Thus, we speculate that defective PI3K-dampening by Itpkb or other moderators contributes to the frequent HSC-defects in bone marrow (BM) failure syndromes [3].

HSC are pluripotent and long-lived. Their longevity relies on their relative metabolic quiescence and only rare division for self-renewal. This dormancy is facilitated by HSC-residence in hypoxic BM niches. Metabolic adaptation and interactions with niche adhesion-molecules and cyto-/chemokines including stem-cell-factor (SCF) keep HSC quiescent and self-renewing [2, 4]. Hematopoietic stress causes the release of cytokines or other factors which mobilize and activate HSC to proliferate and generate shorter-lived HPC. These give rise to the different blood cell lineages. Once blood cell homeostasis is re-established, HSC re-enter dormancy. This is critical, because continuous HSC-activation reduces self-renewal and ultimately depletes HSC, causing BM failure and immunodeficiencies, and promoting certain blood cancers [1, 2, 4].

Increasing evidence suggests that signaling from cytokines like SCF in dormant HSC must be tuned into an intensity-range that ensures self-renewal, but avoids activation. The underlying mechanisms are incompletely understood [1, 2]. Activated by cytokines and other signals, PI3K and its effectors Akt and mTORC1 are required for HSC self-renewal and function. But excessive PI3K/Akt activity transiently expands HSC, followed by depletion and reduced long-term engraftment associated with variable myeloproliferative disease, T-cell-acute-lymphoblastic (T-ALL) or acute-myeloblastic-leukemia (AML) [1, 2]. PI3K produce the membrane-lipid phosphatidylinositol(3,4,5)trisphosphate (PIP_3_), a recruiting and activating ligand for Akt and other effectors. To prevent excessive PI3K-signaling, PIP_3_-levels in many cell types are limited through its removal by the lipid-phosphatases PTEN and SHIP (references in [1, 2]). But the relative importance of HSC in- *versus* extrinsic PTEN functions remains controversial, and SHIP-1 may primarily control HSC-homeostasis cell-extrinsically (references in [2]). Could there be another HSC-intrinsic PI3K-moderator?

Itpkb phosphorylates the Ca^2+^-mobilizing second-messenger IP_3_ into inositol(1,3,4,5)tetrakisphosphate (IP_4_). We and others have identified receptor-induced IP_4_ production by Itpkb as essential for signaling in lymphocytes, granulocyte-monocyte-progenitors (GMP) and neutrophils (references in [2, 5]). IP_4_ is identical with the protein-binding moiety of PIP_3_. In NK cells, GMP and neutrophils, IP_4_ competitively limits PIP_3_-binding to and activation of Akt (references in [2]). ItpkB is expressed in HSC. To thus determine if IP_4_/PIP_3_-antagonism moderates PI3K-signaling in HSC, we studied *Itpkb^−/−^* mice. Young *Itpkb^−/−^* mice accumulated phenotypic HSC which were less quiescent and proliferated more than *Itpkb^+/+^* controls [2]. Transcriptome-analyses showed downregulation of stemness- and quiescence-associated, but upregulation of activation- and differentiation-associated genes in *Itpkb^−/−^ vs*. *wt* HSC. *Itpkb^−/−^* HSC showed intact BM-homing, but reduced *in vitro* persistence and colony-forming-unit (CFU) activity. They had severely reduced competitive long-term-repopulating potential. Aging *ItpkB^−/−^* mice lost HSC and HPC and died with anemia.

**Figure 1:**
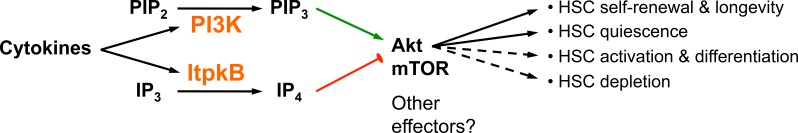
Symmetric signaling by PI3K and Itpkb through the antagonistic second messengers PIP_3_ and IP_4_ restricts signaling by Akt and downstream mTORC1 in HSC into a window that ensures self-renewal, quiescence and longevity but prevents activation, differentiation and eventual depletion [2].

Supporting an Itpkb-role in moderating PI3K-signaling in HSC, *Itpkb^−/−^* HSC had elevated mTORC1 activity *in vivo*, and showed increased SCF-activation of Akt and mTORC1 *in vitro*. This was prevented by treatment with cell-permeable IP_4_ or an Akt-inhibitor. Transcriptome-analysis suggested Akt/mTORC1-hyperactivity and increased oxidative phosphorylation and protein biosynthesis in *Itpkb^−/−^* HSC. A recent study suggests that HSC-quiescence requires restrained protein biosynthesis and implies moderation of mTOR signaling [6]. Injection of the mTOR-inhibitor Rapamycin reversed the HSC-hyperproliferation in *Itpkb^−/−^* mice [2]. Thus, we propose that Itpkb limits cytokine and PI3K/Akt/mTOR signaling in HSC to ensure quiescence and longevity (Figure 1).

The HSC transient expansion but later depletion in *Itpkb^−/−^* mice resembles the phenotypes of PTEN-inactivation or dominant-active Akt expression. But *Itpkb^−/−^* mice have not yet shown T-ALL or AML. Possible reasons include differential effects of PTEN-loss, Akt-activation or Itpkb-loss on PI3K-signaling in HSC, or premature death of *Itpkb^−/−^* mice from anemia or infections [2]. Itpkb-loss might also impair PI3K-unrelated signaling events that promote neoplasia. Clarifying whether Itpkb-loss transforms hematopoietic cells will require conditional knockouts and larger aged cohorts. This will also rule out that Itpkb-loss in other cells indirectly contributes to the HSC-activation in *Itpkb^−/−^* mice [5].

Interestingly, Rapamycin reversed the HSC-hyperproliferation in *Itpkb^−/−^* mice but did not rescue their CFU-activity. This contrasts with *PTEN^−/−^* or *myrAkt*-expressing HSC and may suggest that mTORC1-unrelated mechanisms contribute to HSC-control by Itpkb [2]. But Rapamycin also reduced *wt* HSC CFU-activity, and genetic studies suggest mTORC1-requirements for HSC-regeneration and -function (references in [2]). This might explain the difficulty of rescuing *Itpkb^−/−^* HSC-function with mTORC1-inhibitors, and raises concerns that long-term therapeutic mTORC1-inhibition to prevent aging [7] might damage HSC.

Wrapping up, Itpkb-identification as a moderator of cytokine- and PI3K-signaling in HSC advances our understanding of the HSC-intrinsic mechanisms balancing self-renewal with activation and broadens the importance of IP_4_/PIP_3_-antagonism as a non-canonical mechanism regulating PI3K-function. It will now be important to assess the human relevance of this mechanism, and to determine if human BM failure patients show disease-driving mutations in *Itpkb*. Moreover, it will be interesting o explore if transient specific and selective Itpkb-inhibition can mobilize or expand human HSC without impairing their function.
